# SARS-CoV-2 and Influenza Co-Circulation and Co-Vaccination: A Narrative Review

**DOI:** 10.3390/vaccines14030283

**Published:** 2026-03-23

**Authors:** Mohammad Kamransarkandi, Elena A. Varyushina, Andrey N. Gorshkov, Marina A. Stukova

**Affiliations:** Smorodintsev Research Institute of Influenza, Ministry of Health of the Russian Federation, 197022 Saint Petersburg, Russia; mohamadkamran69@gmail.com (M.K.); marina.stukova@influenza.spb.ru (M.A.S.)

**Keywords:** SARS-CoV-2, influenza virus, co-circulation, co-infection, simultaneous vaccination, next-generation vaccines

## Abstract

Background/Objectives: Severe acute respiratory syndrome coronavirus 2 (SARS-CoV-2) and influenza virus are dangerous respiratory pathogens with high pandemic potential. Since 2021, these two viruses have been co-circulating, which implies additional risks of co-infection with both pathogens. Prophylactic vaccination is widely recognized as the most effective way to prevent COVID-19 and influenza and to reduce the severity of these diseases. This review analyzes recent data on the simultaneous circulation of influenza and SARS-CoV-2 viruses worldwide, including epidemiological data and the pathogenetic mechanisms of co-infection. Next, we focus on current approaches to simultaneous and combined vaccination against influenza and COVID-19. We outline the types of vaccines and summarize the available findings on the effectiveness and safety of co-vaccination. Methods: A comprehensive search was conducted using PubMed, Scopus, Web of Science, and ClinicalTrials to identify data relevant to SARS-CoV-2 and influenza co-circulation and dual vaccination. Results: Influenza and SARS-CoV-2 cause similar symptoms, and co-infection can significantly enhance the risks of pneumonia and acute respiratory distress syndrome progressing with a poor outcome, especially among children and the elderly. A range of influenza and COVID-19 vaccines built on different technological platforms is currently available on the market, with proven effectiveness, immunogenicity, and safety. A co-vaccination approach is more convenient for patients and is associated with better response to treatment, while also improving vaccine coverage and compliance and offering significant resource savings for healthcare systems. Conclusions: The concurrent circulation of SARS-CoV-2 and influenza viruses presents a growing public health challenge. Simultaneous and combination vaccination strategies have emerged as effective tools to streamline immunization, enhance protection, and reduce healthcare burden. Future studies should elucidate the mechanisms of the exacerbation of respiratory disease caused by co-infection, as well as the optimal strategies for co-administering influenza and COVID-19 vaccines for long-term control of seasonal and potentially pandemic respiratory viruses.

## 1. Introduction

Influenza A virus (IAV) is a widespread and dangerous pathogen that causes seasonal epidemics and periodic global pandemics with serious medical and socio-economic consequences [1]. Seasonal epidemics are associated with the circulation of IAV subtypes H1N1 and H3N2. Each year, influenza causes between 3 and 5 million cases of severe illness and 290,000 to 650,000 deaths from respiratory causes [2]. Of particular concern to the WHO is the risk of zoonotic transmission to humans of other IAV subtypes, primarily the highly pathogenic H5N1 avian influenza, clade 2.3.4.4 b, due to the high mortality rate during human infection and the significant pandemic potential of this pathogen [3].

In addition to the flu, in recent years the world has faced the emergence of the SARS-CoV-2 virus and the unprecedented COVID-19 pandemic. According to WHO data, from 31 December 2019 to 7 December 2025, 778,994,897 cases of COVID-19 in humans were officially confirmed worldwide, including 7,106,996 COVID-19 deaths (0.9% of the number of cases) [4]. The European region ranks first among the regions of the world in terms of the total number of detected cases. In Russia, from 3 February 2020 to 5 May 2024, 24,184,485 cases of COVID-19 were registered in 85 regions of the country [5]. The COVID-19 pandemic has had a dramatic negative impact on the global economy and health systems around the world.

Though the COVID-19 pandemic officially ended in 2023 [6], people continue to encounter this virus. SARS-CoV-2 is evolving, and new contagious strains are emerging, so the healthcare system must be prepared for different scenarios. Apparently, COVID-19 has become a seasonal epidemic, and both influenza and SARS-CoV-2 viruses will continue to represent a significant threat to public health for the foreseeable future. In terms of epidemiology, COVID-19 seems to exhibit seasonal trends similar to influenza. SARS-CoV-2 and influenza viruses can co-circulate in autumn and winter, leading to overlapping outbreaks [7]. The dynamic circulation of influenza viruses in conjunction with SARS-CoV-2 is registered all over the world, including in Russia. This raises concerns about the potential risk of infection by each of these two pathogens or co-infection by both of them, which significantly increases the risk to public health.

Despite the differences in the entry mechanisms into target cells, similar modes of transmission and infection sites in the respiratory tract are characteristic of both viral pathogens [8,9]. Influenza and COVID-19 exhibit a generally similar array of symptoms, such as fever, sore throat, chills, chest and abdominal pain, vomiting, diarrhea, nasal symptoms, and loss of taste [10,11,12]. The simultaneous or sequential presence of these two pathogens in lung tissue enhances the risk of serious complications and aggravation of the clinical picture of the disease. Studies conducted in several countries around the world have documented cases of co-infection with SARS-CoV-2 and influenza viruses of types A or B [13]. Co-infections cause reasonable concern among specialists, as they can worsen the course of the disease and introduce additional difficulties into the treatment process. Co-infection with SARS-CoV-2 and influenza A virus leads to a worsening of respiratory ailments such as pneumonia, sinus infections, and bronchitis and increases the danger of acute respiratory failure and cardiovascular complications [14,15]. The mechanisms that cause a more severe course of the disease in co-infection can be multifactorial, including an imbalance in immune regulation, which, in turn, can lead to more significant damage to lung tissue and increased vulnerability to the development of acute respiratory distress syndrome [16,17]. Thus, in the case of co-infection, one of the viruses can enhance the pathogenic effects of the other, which makes it difficult to accurately interpret the clinical manifestations of the disease.

Vaccination remains the most effective strategy to mitigate the burden of both influenza and COVID-19. Simultaneous administration of influenza and COVID-19 vaccines is being actively explored as a way to improve immunization coverage and streamline public health efforts. In October 2021, the WHO approved the combined use of vaccines against COVID-19 and seasonal influenza [18]. The Russian Ministry of Health has also authorized simultaneous vaccination and updated the instructions for use of the vaccine “Sputnik V” [19]. Simultaneous vaccination is the administration of several vaccines on the same clinical day in different anatomical areas, while it is possible to use vaccines with different routes of administration (for example, intramuscularly and intranasally). Clinical trials have further confirmed the feasibility of simultaneous vaccination against influenza and SARS-CoV-2 as an effective approach to immunization [20]. However, it is important to consider such factors as vaccine compatibility, as well as differences in storage requirements and administration schedules.

Another approach is to use combination vaccines, which consist of two or more components that are physically combined and injected simultaneously into the same anatomical site. Combined vaccines targeting both SARS-CoV-2 and influenza are an important area in the new vaccines’ development. With that, it should be considered that the combination of several vaccines can affect the immunogenic properties of individual antigens. Simultaneous vaccination or the use of combined vaccines is more economical, as well as more convenient for patients. A number of vaccines under development have confirmed their immunogenicity in preclinical trials, and several are undergoing clinical trials.

Our review analyzes recent data on the co-circulation of influenza and SARS-CoV-2 viruses worldwide. We highlight the pathogenetic mechanisms of co-infection with these two viruses. Next, we explore the rationale, current evidence, and future potential of simultaneous and combination vaccination strategies targeting influenza and SARS-CoV-2 as an effective approach to immunization. We outline the types of vaccines on various platforms and summarize the available findings on the effectiveness and safety of co-vaccination. Despite the rapidly expanding body of literature addressing SARS-CoV-2 and influenza, an integrated and comprehensive synthesis of their simultaneous circulation, clinical interaction, diagnostic challenges, vaccination strategies, and next-generation vaccine development remains limited. Consequently, important conceptual and practical gaps persist regarding optimal surveillance strategies, diagnostic algorithms, vaccination policies, and preparedness planning in the era of sustained co-circulation. In this regard, our review aims to offer a multidimensional synthesis of current evidence on SARS-CoV-2 and influenza co-circulation, co-infection, and co-vaccination. By integrating clinical data, public health policy, immunological mechanisms, and emerging vaccine technologies, we seek to provide a consolidated perspective that informs clinical decision-making, supports evidence-based public health strategies, and guides future research and vaccine development efforts. A comprehensive literature search was conducted using PubMed, Web of Science, Scopus, and https://clinicaltrials.gov/. Searches were performed using combinations of the following keywords: SARS-CoV-2, influenza virus, co-circulation, co-infection, vaccine co-administration, combined vaccine, vaccine immunogenicity, vaccine safety, and next-generation vaccines. The search was limited to articles published in English up to the time of manuscript preparation and revision in 2026. Original research articles, clinical trials, observational studies, systematic reviews, meta-analyses, and relevant public health reports were considered eligible if they addressed at least one of the following topics: (i) epidemiology and clinical outcomes of SARS-CoV-2 and influenza co-infection; (ii) diagnostic strategies for simultaneous detection of respiratory viruses; (iii) immunological mechanisms underlying co-infection or co-vaccination; (iv) safety, immunogenicity, and effectiveness of simultaneous or combined vaccination against COVID-19 and influenza; and (v) development of combined or next-generation vaccines targeting both viruses.

Publications were excluded if they were unrelated to respiratory viral co-infection, lacked primary data or clinical relevance, were limited to in vitro experiments without translational implications, or did not provide sufficient methodological detail. Additional relevant articles were identified through manual screening of reference lists from key publications.

## 2. SARS-CoV-2 and Influenza Virus Co-Circulation and Co-Infection

Continuous monitoring of circulating respiratory pathogens, including influenza and SARS-CoV-2 viruses, is provided by the global genomic surveillance system. In recent years, with cost reduction and extensive implementation of next-generation sequencing (NGS) technologies, genomic surveillance has rapidly progressed from a specialized research tool to a foundational mechanism for real-time pathogen tracking and public health decision-making. However, its deployment across the globe is still very inconsistent, due to persisting regional differences in the availability of high-throughput sequencing and data sharing. Genomic surveillance has yet to be optimally used to ensure pandemic and epidemic preparedness and response. It is necessary to coordinate resources, use existing systems and assets to strengthen the collective global health security [21]. Timely monitoring of virus activity and evolution helps the WHO to guide countries in preparedness planning, vaccine policy, and resource allocation. The information is shared through FluNet and FluID by the Global Influenza Surveillance and Response System (GISRS) and national epidemiological institutions [22]. Between 2019 and 2025, the global landscape of genomic surveillance expanded significantly in response to COVID-19 and the threat of novel influenza strains. International collaborations such as GISAID (Global Initiative on Sharing All Influenza Data), which began as a platform for influenza viruses, became the world’s most widely used repository for SARS-CoV-2 genome sequences [23]. Although the COVID-19 pandemic officially ended in 2023, SARS-CoV-2 viruses continue to circulate as a seasonal threat, similar to influenza and other acute respiratory viral infections. SARS Cov-2 evolution does not stop; significant genetic and antigenic changes are detected constantly. According to WHO data, as of January 2026, Omicron sub-lineages are the main circulating strains in the world, including Stratus variant (XFG) 83.3%, JN1 2.08%, NB1.8 2.08% and others 12.5% [24]. These strains exhibited enhanced transmissibility and a greater capacity to evade immunity acquired through prior vaccination or infection.

**Figure 1:**
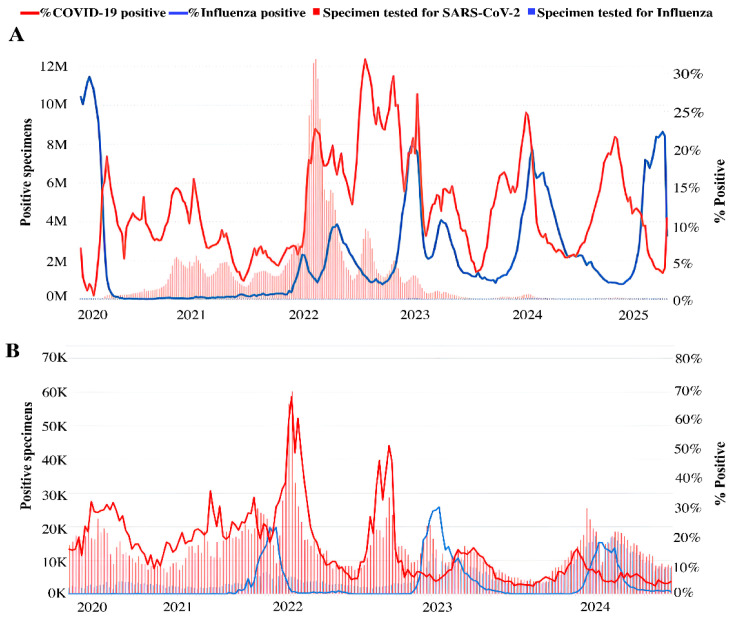
Epidemiological situation of COVID-19 and influenza from 2020. (**A**) Epidemiological situation of COVID-19 and influenza in the world, adapted from World Health Organization (WHO) 2025, Influenza and SARS-CoV-2 virus detections reported to FluNet [25]; (**B**) Circulation of influenza and SARS-CoV-2 viruses in Russia based on data from the A.A. Smorodintsev Influenza Research Institute. Modified from [26].

Along with SARS-CoV-2 monitoring, WHO continues to coordinate global genomic surveillance of influenza, which remains a major public health threat. After a period of practical disappearance from circulation in 2020 and the first half of 2021, influenza viruses resumed circulation at the end of 2021 and, according to epidemiological data, continue to show pronounced genetic variability. As per surveillance data, during the COVID-19 pandemic, the circulation of influenza viruses is reported worldwide (Figure 1A) [25], including in Russia (Figure 1B) [26].

In routine practice of clinical diagnostics, multiplex real-time reverse transcription polymerase chain reaction (RT-PCR) assays have become the standard for the simultaneous detection of SARS-CoV-2, influenza A and B viruses, and, in many platforms, additional respiratory pathogens such as respiratory syncytial virus (RSV) [27,28,29]. These assays enable rapid, sensitive, and specific differential diagnosis of acute respiratory infections presenting with overlapping clinical manifestations, thereby supporting timely clinical decision-making, appropriate antiviral therapy, optimized patient triage, and effective infection control measures. Furthermore, their use supports real-time epidemiological surveillance and improves outbreak detection, contributing to a more accurate assessment of disease burden and transmission dynamics during periods of co-circulation. The integration of multiplex molecular diagnostics into routine clinical workflows, therefore, represents a critical component of current strategies for managing concurrent influenza and COVID-19 circulation, including in the post-COVID era [30].

To investigate the impact of SARS-CoV-2 on influenza activity worldwide, WHO global surveillance data [31,32] were analyzed to compare the number of cases of COVID-19 and influenza in 22 different countries. In that work, Takashita et al. claimed that though both viruses co-circulate, there is rather an alternating pattern in the prevalence of SARS-CoV-2 and influenza virus during the reported period [33].

The dynamic circulation raises concerns about the risk of infection with either of these two pathogens, as well as the risk of co-infection with two viruses. Vulnerable groups such as pediatric and elderly cohorts are at higher risk of infections. Children and elderly individuals exhibit higher susceptibility and face significantly worse clinical outcomes, as well as the disproportionate burden of co-infection rates of influenza and COVID-19 compared to younger adults [34,35,36]. This increased vulnerability is largely attributed to multiple age-dependent immune factors, including immature immune responses in children and immune senescence in older adults, which affect antiviral defenses at various levels, such as mucociliary clearance, epithelial barrier integrity, innate immune activation, adaptive immune responses, and inflammatory regulation [37,38,39]. More specifically, the function of macrophages and neutrophils is decreased in children and the elderly, which impairs phagocytosis, clearance and antiviral reactions. Plasmacytoid dendritic cells produce less interferons of type I and type III. In both age groups, there is an imbalance in the cytokine profile, which leads to a disturbance of the antiviral immune response. In infants, immature alveolar macrophages produce low levels of TNF-α and IL-1, which are necessary for virus clearance. In the elderly, in contrast, production of pro-inflammatory cytokines such as IL-6, IL-8, and TNF-α increases with age (phenomenon of “inflammaging”), which causes higher inflammatory response and, in turn, could increase the severity of disease. This imbalance in cytokine production interferes with the tissue repair mechanisms that follow viral infections. In newborns, natural killer cells (NK cells) have an innate cytotoxicity dysfunction. The elderly have an age-related accumulation of abnormal NK cells, with abnormalities in both cytokine secretion and cytotoxicity of target cells. In both age cohorts, T-cell dysfunction worsens adaptive antiviral responses [40]. At an early age, there is a shift in the response from Th1 towards Th2. In addition, infants’ T cells have a limited ability to form memory. In the elderly, antiviral response is not sufficient due to the aging of antigen-presenting T cells. Overall, these findings reinforce the importance of specific targeted clinical management in these high-risk age groups to reduce disease burden, complications, and mortality.

In the context of co-circulation, it is important to understand the mechanisms of interaction between SARS-CoV-2 and influenza viruses as well as the consequences of such concomitant infections. In general, competitive interference has been described in the case of co-circulation between respiratory viruses, notably influenza [41]. Possible mechanisms underlying these processes can be associated with competition of viruses for host cell resources and the activation of the immune response [42,43,44]. Studies have indicated a significant contribution of the innate immune response to inter-viral interactions, especially when co-infections occur simultaneously or consistently close in time [44,45].

An in vitro approach using the human epithelial airway cells has shown that in the case of sequential infection with SARS-CoV-2 and influenza, respiratory syncytial virus (RSV), or rhinovirus, the type of virus and the order of infections are crucial factors in virus-virus interactions. In this regard, primary infection plays a significant role in shaping the immune response and influencing the outcome of subsequent infections [45]. Pinky et al. created a mathematical model of co-infection with two viruses and showed that SARS-CoV-2 has a lower rate of spread than influenza and can be suppressed if infections start simultaneously. However, if influenza infection occurs later than SARS-CoV-2 infection starts, co-infection may appear [46].

The results of a recent study have shown that the influenza A virus can promote SARS-CoV-2 virus infectivity due to an increase in the expression of angiotensin-converting enzyme 2 (ACE2), allowing the SARS-CoV-2 virus to enter the cells [47]. In addition to the similarities in symptoms between the two infections, SARS-CoV-2 specifically infects type II alveolar cells (AT2 pneumocytes), which are also the site of IAV replication [48,49]. The study by Ziegler et al. analyzed single-cell RNA sequencing data from human lung explants infected with the influenza A virus ex vivo for 24 h. The data obtained established that ACE2 is expressed within type II pneumocytes. Furthermore, after IAV infection, ACE2 expression was elevated [50]. Such elevation of ACE2 expression and of SARS-CoV-2 infectivity is characteristic only of IAV, but not of other respiratory viruses [47].

In animal models, co-infection with influenza A/H1N1 and SARS-CoV-2 viruses clearly prolongs COVID-19 symptoms, causes more severe and prolonged pneumonia, and increases lung damage and weight loss, leading to high mortality [51,52,53].

Clinically, co-infection of the SARS-CoV-2 and influenza A viruses was first reported in a Chinese patient suffering from pneumonia in 2020 [54]. Later, numerous case reports on the co-infection from medical centers around the world were published. These results were summarized in several informative meta-analytical reviews [13,55,56]. According to the results of a recent comprehensive meta-analysis by Golpour et al. [13], the average prevalence of influenza A/B co-infection in COVID-19-positive patients was 14 percent, which is certainly a significant value that requires the careful attention of medical personnel.

Numerous clinical observations clearly indicate that SARS-CoV-2 and influenza co-infection has been associated with significantly worse outcomes, posing a serious public health challenge [32,57]. Given the concurrent circulation, in terms of diagnostics, it is important to consider the COVID-19 co-infection possibility, even if other respiratory viral pathogens have been identified. Particularly, Ma et al. found that a significant number of critically ill COVID-19 patients were co-infected with influenza, potentially leading to earlier cytokine storms and organ damage [17]. Studies have shown that influenza and SARS-CoV-2 co-infection distinctly contribute to the severe form of the disease and, as a result, increase mortality rates [38,58].

Collectively, the available clinical, epidemiological, and immunological evidence supports the concept that co-circulation of SARS-CoV-2 and influenza represents a persistent and evolving public health challenge consistently associated with increased disease severity, higher hospitalization rates, and elevated mortality underscores its clinical relevance, particularly among vulnerable populations. The convergence of diagnostic complexity, overlapping symptomatology, and seasonal epidemic patterns highlights the necessity for integrated surveillance systems and multiplex diagnostic approaches. Simultaneous or combined vaccinations are important strategies for preventing influenza and COVID-19, reducing the risk of infection and co-infection, and minimizing the burden on medical care.

## 3. Concepts of Simultaneous and Combined Vaccination Against COVID-19 and Influenza

Simultaneous vaccination is the administration of multiple vaccines on the same clinic day at different anatomic sites, aiming to induce immune responses to each vaccine while reducing the burden of multiple visits [59,60]. Simultaneous vaccination offers several benefits, including convenience, improved coverage, enhanced compliance, and cost-effectiveness. It reduces the number of required visits, diminishes missed doses, and improves adherence to recommended immunization schedules [61,62]. Additionally, simultaneous vaccination may increase vaccine uptake without compromising the effectiveness of either vaccine [63]. In response to the increasing co-circulation of SARS-CoV-2 and influenza viruses, major international public health authorities have issued explicit guidance supporting the simultaneous administration of COVID-19 and seasonal influenza vaccines. The United States Centers for Disease Control and Prevention (CDC) states that COVID-19 vaccines may be administered without regard to the timing of other vaccines, including seasonal influenza vaccines, enabling same-day co-administration at different anatomical sites to improve vaccine uptake and programmatic efficiency [64]. Similarly, the World Health Organization (WHO) supports the concomitant administration of COVID-19 vaccines with other inactivated vaccines, including influenza vaccines, based on established immunization principles and available safety and immunogenicity data. Together, these recommendations provide a strong regulatory and clinical framework for the implementation of combined and simultaneous vaccination strategies [18]. Accordingly, the integrated genomic surveillance plays the critical role in the annual strain selection for co-administered and combination vaccines, in order to enhance vaccine-induced immune responses to circulating SARS-CoV-2 and influenza variants. Currently, the WHO Technical Advisory Group on COVID-19 Vaccine Composition (TAG-CO-VAC) advises manufacturers that monovalent JN.1 or KP.2 vaccines remain appropriate vaccine antigens, while LP.8.1 is a suitable alternative vaccine antigen [65].

However, when considering simultaneous vaccination, healthcare professionals should account for various factors, such as vaccine compatibility, the individual’s health status, and differences in storage requirements and administration schedules.

Another strategy is to use combination vaccines, which consist of two or more vaccines physically combined and administered simultaneously at the same anatomic site. Combination vaccines offer additional advantages such as reduced storage and handling requirements, increased vaccine efficacy, and convenience for patients [66,67]. Administration of combination vaccines may be more effective in reducing the overall number of injections required, which can be particularly important for patients with needle phobia or anxiety [61,68,69].

**Figure 2:**
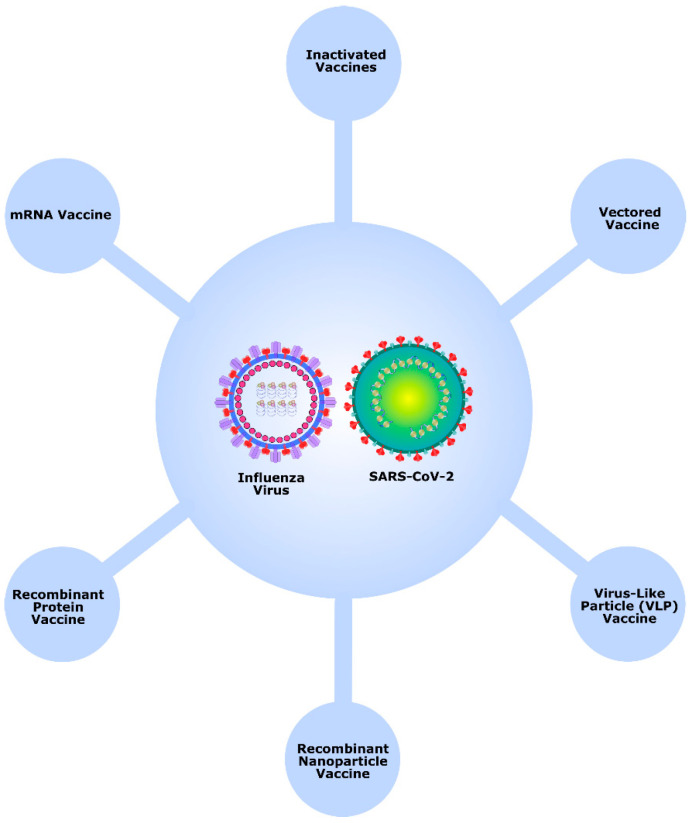
Main biotechnological platforms used in combination vaccines against COVID-19 and seasonal influenza.

To prevent both COVID-19 and seasonal influenza with a single vaccine, the development of combination vaccines targeting these two respiratory diseases has become increasingly relevant. In summary, combination vaccines against COVID-19 and seasonal influenza employ various biotechnological platforms (Figure 2). Inactivated vaccines use killed viruses to stimulate immunity; vectored vaccines deliver genetic material via harmless viruses; virus-like particle vaccines mimic viral structures to provoke an immune response; recombinant nanoparticle vaccines present antigens using engineered nanoparticles; recombinant protein vaccines introduce recombinant viral proteins; and mRNA vaccines instruct cells to produce viral antigens. Each platform offers distinct approaches to generating protective immunity against both diseases.

However, combining multiple vaccines may interfere with the immunogenic properties of individual antigens and complicate vaccination schedules [70,71,72,73].

Therefore, extensive research is needed on the safety and efficacy of simultaneous and combination vaccination, as well as on the characteristics of the post-vaccination immune response in experimental and clinical studies.

## 4. Animal Studies of Co-Vaccination Against COVID-19 and Influenza

Co-vaccination against influenza and COVID-19 with vaccines developed on different technological platforms has been repeatedly researched in relevant animal models, such as K18-hACE2 mice and hamsters.

The effectiveness of the co-vaccination against influenza A (H1N1) and SARS-CoV-2 was investigated using the transgenic K18-hACE2 mouse model. This study assessed the efficacy of the PiCoVacc SARS-CoV-2 vaccine, the flu vaccine, and a co-vaccination in K18-hACE2 mice, using various infection and vaccination groups. The results indicated that mice infected with H1N1 experienced significant weight loss and viral loads, while those infected with SARS-CoV-2 did not survive the observation period. Mice with sequential infection with H1N1 followed by SARS-CoV-2 also showed accelerated mortality due to compromised immune responses. In terms of vaccination, mice that received only the PiCoVacc SARS-CoV-2 vaccine exhibited a strong neutralizing antibody response to SARS-CoV-2, while mice immunized with the H1N1 flu vaccine alone showed a positive response to the flu vaccine. The combined vaccination group demonstrated neutralizing antibodies for both viruses, with levels comparable to single vaccines. It can be concluded that simultaneous vaccination against H1N1 and SARS-CoV-2 provides effective protection against both infections [53].

An inactivated virus-based vaccine targeting both COVID-19 and influenza was studied by Singh et al. [74]. This combination vaccine encapsulated inactivated whole viruses of SARS-CoV-2 (Delta and Omicron variants) and Influenza A (H1N1 and H3N2 strains) within biodegradable PLGA polymer microparticles, formulated with the AddaVax™ adjuvant. Administered to mice via the intranasal route, this microparticulate system aims to provide sustained antigen release and target the mucosal immune system at the primary site of infection. The vaccine successfully induced a robust dual immune response, generating significant levels of virus-specific IgG and mucosal IgA antibodies, alongside strong activation of CD4+ and CD8+ T-cells. The immune response elicited was comparable to that achieved by traditional intramuscular vaccination, supporting the potential of this intranasal combination vaccine as a convenient and effective strategy for simultaneous protection against both respiratory pathogens [74].

Chaparian et al. developed a chimeric influenza virus that simultaneously displays influenza HA and the SARS-CoV-2 RBD. In prime-boost mode, this vaccine was successfully used in mice in live attenuated (prime vaccination) and inactivated (boost vaccination) form. Vaccination with this combination vaccine elicited neutralizing antibodies and provided protection from lethal challenge with both influenza and SARS-CoV-2 [75].

Wang et al. developed a virus-like particle (VLP) vaccine by conjugating the recombinant RBD of the SARS-CoV-2 spike protein onto an inactivated influenza A virus. The resulting vaccine, Flu-RBD, induced protective immunity against SARS-CoV-2 while retaining functionality as an influenza vaccine. In a hamster model, the vaccine conferred protection against live SARS-CoV-2 infection. It exhibited strong neutralization activity against both the SARS-CoV-2 Delta pseudovirus and the wild-type influenza A (H1N1) inactivated virus in mice [76].

Recently, another VLP-based combination vaccine candidate was created by Sanchez-Martinez et al. [77]. VLPs produced in CHO cells contained full-length SARS-CoV-2 S-protein, influenza H1N1 hemagglutinin, and neuraminidase (S + H1 + N1) incorporated into the VLPs’ envelope. The authors demonstrated that two doses of trivalent VLPs elicit specific antibodies and cellular immunity in a mouse model.

Shi et al. developed a combination liposome-enclosed vaccine candidate by mixing recombinant RBD-trimer and HA1-trimer, which conferred protection against SARS-CoV-2 and a lethal homologous H1N1 influenza challenge. Indeed, the RBD-trimer elicited significantly higher neutralizing antibody titers compared to the RBD-monomer, RBD-dimer, and spike ectodomain trimer. Additionally, the vaccine induced a balanced T helper cell (Th1/Th2) cellular immune response in mice [78].

Huang et al. designed a Flu-COVID combo vaccination using the AddaVax adjuvant, including the influenza virus hemagglutinin and SARS-CoV-2 spike proteins. This vaccine effectively protected mice from both influenza and SARS-CoV-2 challenges by preventing weight loss and disease progression, eliciting protective immune responses comparable to monovalent influenza or COVID-19 recombinant protein vaccines [79].

Flu-COVID pentavalent recombinant protein-based vaccine was developed by Krasilnikov and co-authors [80]. This vaccine contained SARS-CoV-2 RBD fused with the Fc fragment of the human IgG and HAs of four influenza viruses: A/H1N1-pdm09, A/H3H2, B/Yamagata, and B/Victoria. Betulin was used as an adjuvant to enhance the vaccine’s immunogenicity. In a mouse model, vaccination provided high titers of specific antibodies to all antigens administered in the vaccine, as well as SARS-CoV-2 and influenza virus neutralization.

Several combined mRNA-based candidate vaccines for COVID-19 and influenza were developed. A combined mRNA vaccine (AR-CoV/IAV) for COVID-19 and influenza was developed using a lipid nanoparticle-encapsulated mRNA platform (LNP-mRNA) [81]. This vaccine encodes IAV-HA and SARS-CoV-2-RBD and elicits robust hemagglutination inhibition (HAI) antibodies against IAV, as well as neutralizing antibodies against SARS-CoV-2. It also protects mice from co-infection with IAV and the SARS-CoV-2 Alpha and Delta variants. Moreover, this vaccine t induced Th1 cytokine-secreting CD4+ T cells and interferon gamma (IFN-γ+) or tumor necrosis factor-alpha (TNF-α+) CD8+ T cells, demonstrating enhanced antiviral activity without causing severe disease [81].

FLUCOV-10 is a LNP-mRNA vaccine that encodes full-length hemagglutinin proteins from four seasonal influenza viruses (A/Wisconsin/588/2019 (H1N1) pdm09, A/Darwin/6/2021 (H3N2), B/Austria/1359417/2021, and B/Phuket/3073/2013), two avian influenza viruses posing potential pandemic risks (A/Thailand/NBL1/2006 (H5N1) and A/Anhui/DEWH72-03/2013 (H7N9)), and spike proteins from four SARS-CoV-2 variants (ancestral SARS-CoV-2, BQ.1.1, BA.2.75.2, and XBB.1.5 omicron variants). It has been shown to elicit robust immune responses in mice, including the production of immunoglobulin G (IgG), neutralizing antibodies, and antigen-specific cellular responses against all vaccine-matched influenza and SARS-CoV-2 viruses, as well as complete protection in mouse models against both homologous and heterologous strains of influenza and SARS-CoV-2 [82].

Recently, a study in mice evaluated the co-administration of the seasonal quadrivalent influenza vaccine (QIV) and the Pfizer-BioNTech COVID-19 mRNA vaccine (BNT162b2). The research specifically tested three methods of simultaneous administration: injection into opposite limbs, the same limb, or mixing both vaccines in one syringe. The key finding was that co-administration, particularly when vaccines were given in the same limb or mixed, led to a significantly enhanced antibody response against influenza viruses. Co-administration resulted in a slight reduction in antibody levels against SARS-CoV-2 compared to giving the COVID-19 vaccine alone. Despite this modest reduction, all co-administered regimens provided complete protection, with vaccinated mice showing 100% survival, minimal weight loss, and low viral loads in the lungs after lethal challenge with either virus. This suggests that simultaneous vaccination induces robust protective immunity, though it may differentially modulate the strength of the immune response to each pathogen [83].

A 2025 study developed a novel mRNA-LNP combination vaccine for both influenza and COVID-19. To address the known challenge of low immunogenicity for influenza B strains in mRNA platforms, the researchers used an innovative antigen design. They engineered fusion proteins where hemagglutinin (HA) antigens are linked by a stabilizing bacteriophage T4 foldon domain, creating “dumbbell” or trimeric structures expressed from a single mRNA species. In mice, this combination vaccine elicited hemagglutination inhibition antibody titers against seasonal influenza strains that were significantly higher than those induced by a commercial high-dose inactivated vaccine (Fluzone HD). Simultaneously, it generated superior neutralizing antibody responses against the SARS-CoV-2 XBB.1.5 variant compared to a commercial COVID-19 mRNA vaccine (Spikevax). The study demonstrates a promising antigen design strategy to enhance immunogenicity in multivalent mRNA vaccines [84].

Viral vectors to deliver genetic material encoding target antigens demonstrated good potential for creating new vaccines for respiratory infection prevention [85]. Immunization with vector vaccines elicits strong immune responses against both the vector and the embedded vaccine antigen. Moreover, viral vector vaccines can be easily administered non-invasively, via nasal sprays or nebulization, and are associated with fewer adverse reactions and improved acceptance of vaccines. The administration of intranasal vaccines augments protection against respiratory viruses through the stimulation of the immune system at the primary site of viral infections, thereby fostering a balanced and efficacious immune response. Importantly, immunization with vector vaccines also promotes the production of cytokines and chemokines as part of a defensive inflammatory reaction [86].

Implementation of adenoviral vector vaccines during the COVID-19 pandemic underscored their evident efficacy, favorable safety characteristics, and immunogenicity. Cao et al. described a strategy for developing a chimpanzee adenovirus 68 (AdC68)-based vaccine targeting both SARS-CoV-2 and IAV using a fusion immunogen [87]. It focused on an immunogen created by combining the SARS-CoV-2 receptor-binding domain (RBD) with the conserved stalk of H7N9 hemagglutinin (HA). Ferritin was used as a platform to improve the vaccine’s immunogenicity. The AdC68-CoV/Flu vaccine elicited an antibody response against both viruses. The spike protein elicited strong neutralizing antibody responses against wild-type SARS-CoV-2 strains but lower responses against variants such as Beta (B.1.351, B.1.627) and Gamma (P.1). Extensive RBD-specific T cell responses of splenocytes were revealed. In addition, AdC68-CoV/Flu vaccine provided effective protection against lethal SARS-CoV-2 challenge in hACE2-C57BL/6 mice.

More recently, another new AdC68-HATRBD vaccine was developed using the same AdC68 vector [88]. This vaccine encoded two RBD dimers from various SARS-CoV-2 variants, namely Beta-Alpha chimeric dimer and Omicron-Delta chimeric dimer, as well as numerous T cell epitopes of SARS-CoV-2 and full-length HA of A/California/07/2009 (pH1N1). When administered intranasally, the AdC68-HATRBD vaccine provided comprehensive immune responses, including IgG, mucosal IgA, and memory T cell responses, which protected the mice from BA.5.2 and pandemic H1N1 infections.

The influenza vector platform is based on integrating foreign proteins into attenuated or replication-deficient influenza viruses for developing vaccines targeting various human respiratory pathogens [89,90,91,92,93,94].

Delta-19 is a nasal spray vaccine designed to protect against both COVID-19 and influenza. It is built on Delta NS1 vaccine vector technology, which expresses key immunogenic proteins of both viruses. According to the developer’s (Vivaldi Biosciences) information, the vaccine is currently undergoing challenge-protection studies in animal models and is being prepared for an Investigational New Drug (IND) application and clinical trials [92].

A study by Sergeeva et al. investigated the effectiveness of an intranasal vaccine using a modified influenza vector that encodes the nucleoprotein (N protein) of SARS-CoV-2 and has a truncated NS1 gene, designed to stimulate a robust local immune response, particularly targeting essential CD8+ T-cells [93]. The study revealed that intranasal immunization with the influenza vector significantly reduced weight loss and viral load in the lungs of naïve mice after exposure to the SARS-CoV-2 beta variant, indicating the vaccine’s effectiveness in providing protection in previously unexposed individuals. Additionally, in seropositive Th2-prone mice that had been primed with alum-adjuvanted inactivated SARS-CoV-2, a single intranasal boost with the vaccine was able to prevent disease enhancement, such as early weight loss and eosinophilia in the lungs during infection. The vaccine successfully modulated the immune response to mitigate these symptoms. Overall, the findings highlight that intranasal immunization with influenza vector-based SARS-CoV-2 vaccine holds significant potential for preventing COVID-19 and associated immunopathology, though the direct anti-influenza protective effect of the vaccine has not been assessed in this work [93].

During development of the Pneucolin dNS1-RBD vector vaccine, the nonstructural-1 (NS1) gene of influenza was replaced with the SARS-CoV-2 RBD region. Studies in hamsters have shown that this nasal vaccine stimulated both systemic and local immune responses and preserved body weight after challenge. Moreover, the vaccine provided cross-protection against H1N1 and H5N1 influenza, as well as protection against various variants of SARS-CoV-2. Attenuating pro-inflammatory cytokine levels post SARS-CoV-2 challenge was registered, thereby reducing excess immune-induced lung tissue injury [94,95].

Loes and co-authors developed an influenza virus vector where the SARS-CoV-2 RBD region was inserted in place of the neuraminidase coding sequence. It was shown that the vector vaccine candidate induces robust levels of serum antibodies that are capable of neutralizing both influenza and SARS-CoV-2 viruses [96].

Stepanova and colleagues designed a 3×LAIV/CoV-2 vaccine by modifying a licensed seasonal trivalent live attenuated influenza vaccine. The modification included two LAIV strains, H1N1 and H3N2, encoding a cassette enriched with immunogenic conserved T-cell epitopes of SARS-CoV-2. The third vaccine component, B/Victoria lineage LAIV strain, remained unmodified. In viral challenge experiments, this vaccine exhibited efficient protection against either influenza strain as well as against SARS-CoV-2 challenge. Given these results, 3×LAIV/CoV-2 can be considered as a prospective vaccine candidate for combined prevention of two respiratory diseases, seasonal influenza and COVID-19 [97].

Thus, the results of animal studies demonstrate that simultaneous vaccination and combination vaccines developed using different platforms are effective and immunogenic against SARS-CoV-2 and influenza. These vaccination strategies provide effective protection against both infections.

Animal studies of co-vaccination against SARS-CoV-2 and the influenza virus are summarized in Table 1.

## 5. Clinical Studies of Co-Administration of Influenza and COVID-19 Vaccines

COVID-19 is now becoming an established seasonal epidemic disease, and its severe public health impact has diminished from the pandemic’s initial phase. Nevertheless, it continues to present a substantial health burden as the respiratory infectious disease is accountable for a number of hospitalizations and intensive care unit admissions, especially in patients from high-risk groups. The possibility of future outbreaks highlights the importance of up-to-date vaccination strategies, which are being developed all over the world. In October 2021, the World Health Organization (WHO) approved co-administration of vaccines against COVID-19 and seasonal influenza [18]. In addition to international guidance, multiple national public health authorities worldwide have formally endorsed the co-administration of seasonal influenza and COVID-19 vaccines, further supporting this strategy as a global standard of care. Particularly, the Russian Ministry of Health authorized simultaneous vaccination and updated the instructions for the use of the Sputnik V vaccine accordingly [19]. In France, official public health recommendations advise concomitant vaccination for all individuals eligible for both vaccines, regardless of age, starting from the 2025–2026 campaign [98]. Similarly, Canada’s National Advisory Committee on Immunization (NACI) recommends that all seasonal influenza vaccines, including live attenuated formulations, may be administered simultaneously with, or at any interval before or after, COVID-19 vaccines for individuals aged six months and older [99]. In Taiwan, the publicly funded 2025 vaccination program prioritizes high-risk populations while permitting co-administration of influenza and COVID-19 vaccines [100]. In China, health authorities recommend simultaneous administration of COVID-19 and inactivated influenza vaccines for adults, administered in different arms, while advising precautionary intervals for minors [101,102]. Collectively, these policies reflect a broad international consensus supporting the safety, feasibility, and public health value of influenza and COVID-19 vaccine co-administration.

**Figure 3:**
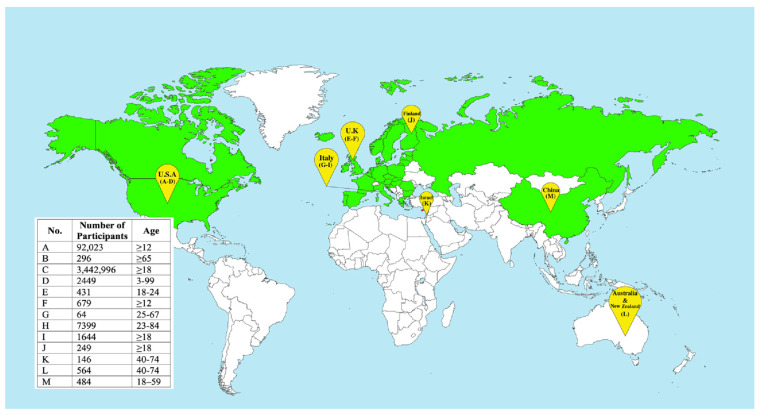
Global participation in the simultaneous vaccination of COVID-19 and influenza. Green: countries that recommended simultaneous use. The table provides data from relevant clinical trials: A (US) [103] B (US) [104] C (US) [105] D (US) [106] E (UK) [107] F (UK) [108] G (Italy) [109] H (Italy) [110] I (Italy) [111] J (Finland) [112] K (Israel) [113] L (Australia and New Zealand) [114] M (China) [115].

During the COVID-19 pandemic, clinical trials have been conducted in several countries, and the results show that simultaneous administration of the vaccines is generally safe and produces comparable reactogenicity and immune responses to separate administration [103,104,105,106,107,108,109,110,111,112,113,114,115]. Additionally, simultaneous vaccination may increase vaccine uptake without compromising the effectiveness of either vaccine [63]. Figure 3 summarizes the geography of these clinical trials and lists the countries that recommend simultaneous vaccination against COVID-19 and seasonal influenza.

Clinical research on the safety and effectiveness of simultaneous vaccination against COVID-19 and influenza is a task of primary importance for public health.

Toback et al. investigated the safety and immunogenicity profiles of NVX-CoV2373 (Novavax; US) vaccinations administered simultaneously with seasonal influenza vaccines. The incidence and severity of local and systemic reactogenicity events after co-administration were generally comparable to those when each vaccine was administered separately. Furthermore, post-vaccination geometric mean titers and seroconversion rates remained high for each strain, regardless of whether the influenza vaccine was administered with placebo or NVX-CoV2373, despite a generally reduced response to the influenza B strains among all influenza vaccine recipients. However, co-administration of NVX-CoV2373 and an influenza vaccine resulted in a modest decrease in anti-spike protein IgG. The levels of anti-spike protein IgG in those who received the two vaccines remained more than threefold higher than those seen in convalescent serum, implying that these levels may be protective [107].

Hause et al. showed that among individuals who received both the seasonal influenza vaccine and either the Pfizer-BioNTech or Moderna COVID-19 mRNA booster simultaneously, systemic reactions were 8–11% more common compared to those who received the COVID-19 booster alone [103].

Izikson et al. conducted a study on the safety and immunogenicity of co-administering a high-dose quadrivalent influenza vaccine (QIV-HD) and an mRNA-1273 vaccine booster dose in older adults. The study found no safety concerns or immune interference in older adults who received a third dose of the mRNA-1273 vaccine with QIV-HD up to 21 days after vaccination. Similar hemagglutination inhibition and SARS-CoV-2 binding antibody responses were observed between the co-administration and QIV-HD groups and between the co-administration and mRNA-1273 groups [104].

A study comparing the effectiveness of co-administering the BNT162b2 BA.4/5 bivalent mRNA COVID-19 vaccine and seasonal influenza vaccines (SIV) in a community setting (which included 3,442,996 commercially insured US adults aged 18 years or older) found that outcomes of co-administration of both vaccines were similar when compared to those of each vaccine against COVID-19 and SIV alone. It suggests that co-administration may improve the uptake of both vaccines [105].

Moro et al. compared reports of systemic reactions and injection site reactions in COVID-19 patients who received a booster dose of an mRNA COVID-19 booster vaccine with a quadrivalent inactivated influenza vaccine (QIV) and those who received a booster dose alone. Systemic reactions were slightly more frequent in reports with the QIV, while injection site reactions and COVID-19 infection were slightly more frequent in reports with only the mRNA COVID-19 vaccine booster dose [106].

The combining influenza and COVID-19 vaccination (ComFluCOV) study was undertaken to determine the side effects, including fever and tiredness, experienced by participants when their second COVID-19 vaccine dose was administered simultaneously with the flu vaccine. In the multicenter, randomized, controlled, phase 4 trial, 679 adult participants were enrolled. They receive a single dose of ChAdOx1 or BNT162b2 and concomitant administration of either an age-appropriate seasonal, inactivated vaccines or placebo alongside their second dose of COVID-19 vaccine. Three weeks later, the group that received a placebo received the influenza vaccine, and vice versa. It was established that most systemic reactions to vaccination were mild or moderate, rates of adverse local and systemic reactions were similar between the groups, and immune responses were not adversely affected. Concomitant vaccination with ChAdOx1 or BNT162b2 plus an age-appropriate influenza vaccine raises no safety concerns and preserves antibody responses to both vaccines [108].

In the study involving 1231 participants, individuals who had received two doses of the BNT162b2 mRNA vaccine followed by a third dose (either BNT162b2 or mRNA-1273) were assessed for co-administration with the influenza vaccine. Co-administration of the influenza vaccination was an option chosen by some of the participants. Anti-SARS-CoV-2-spike IgG levels in the control cohort were significantly higher than in the co-administration group (+34.0%, *p* < 0.01) (median 1605.0 BAU·mL^−1^, interquartile range (IQR) 1341.1–3242.3 BAU·mL^−1^ and median 2150.2 BAU·mL^−1,^ IQR 1078.0–2504.7 BAU·mL^−1^, respectively). Additionally, significantly higher anti-SARS-CoV-2-spike IgG levels were induced by mRNA-1273 vaccination than BNT162b2mRNA (*p* < 0.0001) regardless of whether or not co-administered with the flu vaccine. Although co-administration led to a modest reduction in anti-SARS-CoV-2 spike IgG levels—particularly for mRNA-1273 and BNT162b2, it did not result in increased health-related absenteeism among healthcare workers and thus does not jeopardize public healthcare capacity [112].

A prospective cohort study involving healthcare workers at a large tertiary medical center in Israel assessed the reactogenicity and immunogenicity of co-administration of the Omicron BA.4/BA.5-adapted bivalent COVID-19 vaccine with SIV. Results showed that those who received SIV alone experienced the least reactogenicity, while COVID-19 vaccination alone elicited similar reactogenicity to the co-administration of the vaccine with SIV. Geometric mean titers in the co-administration group were evaluated as 0.84 (95% CI, 0.69–1.04) times lower than in the COVID-19–vaccinated group. The study also revealed a mild 16% decrease in anti-spike IgG titers, which did not significantly impact vaccine effectiveness or protection against symptomatic disease, based on previous studies [113].

A similar study found that co-administering the BNT162b2 vaccine alongside the seasonal inactivated influenza vaccine (SIIV) in adults aged 18–64 was safe and well-tolerated and demonstrated robust immune responses that were not inferior to separate administration of BNT162b2 and SIIV [114].

Moderna successfully conducted Phase 1 and 2 trials to evaluate the safety, reactogenicity, and immunogenicity of mRNA-1073 (SARS-CoV-2 and Influenza Vaccine) when compared to co-administered mRNA-1010 (Influenza) and mRNA-1273 (SARS-CoV-2) vaccines and to mRNA-1010 and mRNA-1273 vaccines in healthy adults 18–75 years [116].

Lee et al. performed a randomized trial that enrolled 56 adults who received the Afluria QIV and the Moderna monovalent SARS-CoV-2 XBB.1.5 mRNA vaccine, either in separate arms or both in the same arm at the same anatomical site. Influenza vaccination’s immunogenicity is comparable regardless of whether it is administered in the same or opposite arms as the SARS-CoV-2 vaccine. However, it may be advisable to administer the SARS-CoV-2 vaccine at a distinct site from influenza vaccines [117].

In a randomized, open-label, controlled study conducted in healthy adults aged 18–59 years in China, the immunogenicity and safety of co-administering the inactivated SARS-CoV-2 vaccine CoronaVac with a quadrivalent inactivated influenza vaccine were evaluated. Co-administration resulted in non-inferior SARS-CoV-2 neutralizing antibody responses compared with sequential vaccination, although modest reductions in geometric mean titers were observed, particularly when influenza vaccination coincided with the second dose of CoronaVac. Importantly, the safety profile remained favorable, with no increase in serious adverse events and only mild-to-moderate local and systemic reactions reported, supporting the clinical feasibility and tolerability of this co-administration strategy [115].

In a randomized controlled trial involving healthy adults, the immunogenicity and safety of co-administering a quadrivalent inactivated influenza vaccine (Afluria) and a SARS-CoV-2 mRNA vaccine (Moderna monovalent XBB.1.5), administered either in the same arm or in opposite arms, were evaluated. No significant differences were observed in HAI titers against influenza A(H1N1), A(H3N2), and B strains between the two vaccination strategies, indicating comparable influenza immunogenicity. Similarly, SARS-CoV-2–specific binding IgG antibody levels and neutralizing antibody titers did not significantly differ between groups following vaccination, although a greater fold increase in neutralizing antibodies against ancestral and BA.5 variants was detected in participants vaccinated in opposite arms. Both administration approaches exhibited comparable local and systemic reactogenicity profiles, with no safety concerns identified [117].

In a large phase 3, observer-blinded, placebo-controlled randomized trial, the safety and immunogenicity of the RSV mRNA-1345 vaccine co-administered with either a seasonal quadrivalent inactivated influenza vaccine (SIIV4) or a bivalent SARS-CoV-2 mRNA vaccine (mRNA-1273.214) were evaluated in adults aged 50 years or older. The study demonstrated that co-administration resulted in reactogenicity profiles comparable to those observed when the vaccines were administered separately, with no vaccine-related serious adverse events reported. Immunogenicity analyses showed mostly non-inferior neutralizing antibody responses against RSV-A, hemagglutination inhibition titers against all four influenza strains, and neutralizing antibody responses against ancestral and Omicron BA.1 SARS-CoV-2 variants. Although a modest reduction in RSV-A seroresponse rate was observed when mRNA-1345 was co-administered with SIIV4, overall immune responses met predefined non-inferiority criteria. These findings support the safety and immunogenicity of co-administering RSV, influenza, and COVID-19 vaccines in older adults and provide strong clinical evidence for integrated respiratory vaccination strategies in high-risk populations [118].

A large population-based retrospective cohort study conducted during the 2023–2024 influenza season in Italy evaluated the effectiveness of quadrivalent inactivated influenza vaccines administered alone or in combination with pneumococcal and/or SARS-CoV-2 vaccines among adults aged 60 years or older. Using comprehensive administrative healthcare databases covering the entire elderly population of the Pescara province, the study demonstrated that influenza vaccination was associated with a significant reduction in both all-cause mortality and hospital admissions due to influenza and/or pneumonia. Notably, co-administration of influenza vaccines with either pneumococcal or COVID-19 vaccines resulted in a further significant decrease in the risk of these adverse outcomes compared to influenza vaccination alone. These findings provide robust real-world evidence supporting the clinical and public health benefits of combined vaccination strategies in older populations and reinforce the role of integrated respiratory immunization programs in reducing severe disease burden and mortality [119].

A phase 3, randomized, observer-blind, multicenter study evaluated the immunogenicity and safety of the self-amplifying mRNA (sa-mRNA) COVID-19 vaccine ARCT-2303, encoding the spike protein of the Omicron XBB.1.5 subvariant, administered alone or concomitantly with seasonal inactivated influenza vaccines in adults. The study demonstrated that ARCT-2303 elicited superior neutralizing antibody responses against Omicron XBB.1.5.6 compared with the ancestral strain vaccine (ARCT-154), meeting predefined superiority criteria. Importantly, co-administration with either standard-dose quadrivalent influenza vaccine in adults aged 18–64 years or adjuvanted influenza vaccine in adults aged ≥65 years did not negatively affect HAI responses to influenza strains. Neutralizing antibody responses against SARS-CoV-2 were non-inferior when vaccines were co-administered compared with separate administration. Reactogenicity and safety profiles were comparable between groups. These findings support the feasibility of co-administering next-generation sa-mRNA COVID-19 vaccines with seasonal influenza vaccines and highlight the potential of self-amplifying mRNA platforms in integrated respiratory vaccination strategies [120].

A phase 3, randomized, double-blind, non-inferiority trial evaluated the safety, reactogenicity, and immunogenicity of concomitant administration of the Ad26.COV2.S COVID-19 vaccine with seasonal quadrivalent influenza vaccines in adults. Participants aged 18–64 years received a standard-dose influenza vaccine, whereas those aged ≥65 years received either standard-dose or high-dose formulations. Co-administration met non-inferiority criteria for SARS-CoV-2 Spike-specific antibody responses and for HAI titres against most influenza strains, with comparable seroconversion and seroprotection rates between co-administered and separately administered groups. Although the predefined non-inferiority margin was narrowly missed for the A/H1N1 strain, overall immune responses were robust and persisted for at least six months. Reactogenicity and safety profiles were consistent with the known profiles of each vaccine, and no safety concerns were identified. These findings support the immunogenicity and tolerability of co-administering adenoviral vector COVID-19 vaccines with both standard-dose and high-dose influenza vaccines across adult age groups [121].

A multisite randomized clinical trial conducted in the United States evaluated whether simultaneous administration of a quadrivalent inactivated influenza vaccine (IIV4) with an mRNA COVID-19 vaccine affected SARS-CoV-2 immunogenicity compared with sequential administration. Participants aged ≥5 years received influenza vaccine or placebo concomitantly with an initial or booster dose of mRNA COVID-19 vaccine, followed by delayed influenza vaccination in the sequential group. Immunogenicity was assessed using pseudovirus neutralization assays against ancestral SARS-CoV-2 (D614G) and Omicron variants (BA.4/BA.5 and XBB.1.5). Post-vaccination geometric mean neutralizing antibody titers did not differ significantly between the simultaneous and sequential groups, indicating no evidence of immune interference or blunting. These findings provide further clinical support for the simultaneous administration of influenza and mRNA COVID-19 vaccines as a practical strategy to improve vaccination coverage without compromising immunogenicity [122].

The recent systematic review summarizes published data regarding the concurrent administration of BNT162b2 mRNA vaccine and licensed seasonal influenza vaccines (SIVs), specifically examining their prevalence, effectiveness, safety profiles, and immunogenicity [123]. The analysis incorporated 15 observational studies and 5 clinical trials. A clear trend showing an increase in the co-administration of BNT162b2 with SIVs over time has been reported, climbing from 2.7% in 2021 to 34.1% in 2023. Importantly, no alteration in effectiveness was observed when BNT162b2 was co-administered with SIVs. Furthermore, the occurrence of both systemic and local adverse events was found to be similar between individuals who received the vaccines separately and those who received them concurrently.

Table 2 represents key parameters of clinical studies of influenza and COVID-19 vaccines co-administration.

Thus, clinical studies of co-administration of vaccines against COVID-19 and influenza proved safety in evaluated scenarios [95,96]. Most side effects were mild to moderate and temporary. While some studies noted a slight increase in reactogenicity, serious adverse events or safety issues were not inherent. The results of the assessment on immunogenicity and correlates of protection vary in published studies of the co-administration of COVID-19 vaccines and SIV. A number of co-administration studies concluded that the humoral response was indistinguishable from that elicited by COVID-19 vaccination alone, confirming clinical non-inferiority of co-vaccination [104,107,108]. With that, some studies reported a mild but statistically significant reduction in post-vaccinal titers or neutralization capacity of anti-SARS-CoV-2-spike antibodies [112,113]. Apparently, in the case of co-vaccination, anti-SARS-CoV-2-spike IgG levels were lower due to simultaneous activation of the immune response to both vaccine antigens.

In general, research on immunogenicity and correlates of protection of COVID-19 vaccines indicates that individual variations in post-vaccinal anti-spike IgG titers did not significantly affect vaccine efficacy. Particularly, they did not alter either the risk of a SARS-CoV-2 infection, nor the disease symptoms severity [124, 125,126]. Strictly validated correlate of protection for SARS-CoV-2 currently still remains undetermined and is actively studied [127]

To summarize, nearly all studies published confirmed that simultaneous vaccination did not significantly alter the immune response for either vaccine. The advantages conferred by vaccine co-administration demonstrably outweigh the associated risks. This emphasizes the benefit of combined administration, potentially improving vaccine acceptance by simplifying immunization procedures and decreasing healthcare visits. However, despite these findings, the optimal approach to co-vaccination against COVID-19 and influenza is the subject of continuous discussion and research [128].

## 6. Clinical Studies of Combined Influenza and SARS-CoV-2 Vaccines

Combination influenza and SARS-CoV-2 vaccines are aimed to provide protection against both infections with a single-dose administration, as a more favorable alternative to simultaneous vaccination with two vaccines. A number of combined vaccines on various biotechnological platforms have proven their immunogenicity and beneficial safety profile in preclinical studies. Several combined vaccines reached clinical trials. A summary of clinical studies on combination vaccines targeting SARS-CoV-2 and influenza is provided in Table 3.

A part of these clinical trials involves the mRNA LNP vaccine platform, which previously became the basis for the globally approved monovalent SARS-CoV-2 mRNA vaccines. mRNA LNP vaccines have a modular technological engineering that allows to rapidly amplify their manufacturing, as well as easily reconfigure the vaccine’s design following the drift in actual viral antigens.

Pfizer and BioNTech launched a Phase 3 trial to evaluate the safety, tolerability, and immunogenicity of a combined modified RNA COVID-19 and influenza vaccine (NCT06178991) [129]. This vaccine elicited superior influenza A and comparable to licensed COVID-19 vaccine immune responses, but it did not achieve non-inferiority against the influenza B strain.

Moderna performed trials of the safety, reactogenicity, and immunogenicity of mRNA-based multicomponent vaccines against seasonal influenza and SARS-CoV-2, namely, mRNA-1073 Phase 1,2 (NCT05375838) [130], mRNA-1083 Phase 1,2 (NCT05827926) [131] and Phase 3 (NCT06097273) [132]. A phase 1 and 2 clinical trial of mRNA-1073 involved comparison of mRNA-1073 against the individual and simultaneous administration of mRNA-1010 (influenza) and mRNA-1273 (SARS-CoV-2) vaccines in overall 550 adults aged 18–75 years (NCT05375838) [130]. The results of safety evaluation of the mRNA-1073 vaccine demonstrated good tolerability, with side effects being mild to moderate and temporary; no serious adverse events related to study vaccination were reported. A single dose of mRNA-1073 elicited potent and balanced antibody responses through 6 months against all vaccine-matched influenza and SARS-CoV-2 strains with comparable immune profiles to mRNA-1010 + mRNA-1273. These findings support continued investigation of single-dose mRNA multicomponent vaccines offering simultaneous protection against seasonal influenza and SARS-CoV-2.

Phase 3 of clinical trials of another multicomponent vaccine, mRNA-1083, involved more than 8000 participants ≥ 50 years of age in two distinct cohorts [132]. The first cohort consisted of 4017 individuals aged 65 and above, compared a single dose of mRNA-1083 against the co-administration of an influenza vaccine, Fluzone HD, and a COVID-19 vaccine, Spikevax. The second cohort encompassed 3998 adults 50–64 years of age, compared a single dose of mRNA-1083 to the co-administration of a standard-dose influenza vaccine, Fluarix and Spikevax. Noninferior immunogenicity of mRNA-1083 was assessed in comparison to existing licensed comparator vaccines. In both age groups, mRNA-1083 demonstrated statistically significant superior immune responses against three specific influenza virus strains (H1N1, H3N2, and B/Victoria) and against SARS-CoV-2 than standard-dose quadrivalent inactivated influenza vaccine SD-IIV4 (50–64 years) and high-dose quadrivalent inactivated influenza vaccine HD-IIV4 (≥65 years). In terms of safety and reactogenicity, mRNA-1083 vaccination showed predominantly mild to moderate (Grade 1 or 2) solicited adverse reactions with a slightly greater incidence compared to comparators across both age groups (≥65 y: 83.5% and 78.1%; 50–64 y: 85.2% and 81.8%). Overall, in this investigation, mRNA-1083 was shown to meet noninferiority criteria and elicited sufficient immune response.

Thus, favorable safety profiles of the multicomponent mRNA vaccines listed above were reported. These vaccines demonstrate the capacity to generate sustained antibody responses against their target pathogens for at least half a year. These data support the use of this immunization strategy for adult populations against seasonal influenza and SARS-CoV-2.

Recently, GlaxoSmithKline initiated a Phase 1,2 clinical trial (NCT06680375) to assess the reactogenicity, safety, and immune response of the mRNA Flu/COVID-19 vaccine [133], with no results reported yet.

Novavax performed a clinical study evaluating the safety and immunogenicity of a SARS-CoV-2 rS nanoparticle and quadrivalent hemagglutinin nanoparticle influenza combination vaccine with Matrix-M adjuvant [134]. This vaccine is designed as adjuvant nanoparticles bearing recombinant antigen proteins of SARS-CoV-2 and the influenza virus. Results of this trial were not published.

In addition to the mRNA LNP and nanoparticle-based combination vaccines listed above, an attenuated influenza virus with a deleted NS segment (delta NS technology) has proven to be a promising biotechnological platform. A significant advantage of intranasal influenza virus vector-based SARS-CoV-2 vaccines is an induction of local immune protection in the upper respiratory tract, and not only systemic immunity. Intranasal vaccines provide additional immune protection via mucosal resident memory B- and T-cells, as well as secretory IgA stimulation. By initiating antiviral effects directly in a potential viral entry point, within the nasal epithelium, the progression of disease can be effectively blocked at an early stage. This is especially important for preventing infections from omicron variants, which have a short incubation period of 2–4 days. Of particular importance, new influenza virus vector-based vaccines generate wide-ranging, robust, and long-lasting immune reactions while maintaining satisfactory safety standards. Additional benefits of intranasal vaccination over injections include its needle-free and non-invasive nature, thereby removing the common pain and fear associated with administration. The broad appeal and simple administration of intranasal vaccination offer potential for reducing vaccine hesitancy and expanding immunization coverage during viral outbreaks, thus easing the disease burden, particularly for vulnerable populations.

Delta NS technology was applied in the development of the dNS1-RBD Pneucolin vaccine with an intranasal route of administration. dNS1-RBD Pneucolin’s efficacy and safety have been demonstrated in several clinical trials (ChiCTR2000037782, ChiCTR2000039715, ChiCTR21000483160) [135,136]. Clinical trials phase 1 and 2, followed by a phase 2 extension trial, were conducted in groups of healthy adults (≥18 years) not previously vaccinated against SARS-CoV-2 [135]. Two doses of dNS1-RBD were well tolerated, with no serious adverse events attributed to the vaccine. T-cell response in peripheral blood was detected in 46% (211/455) of vaccinated participants. RBD-specific IgG seroconversion occurred in 48 of 466 vaccine recipients, with a GMT of 3.8 (95% CI 3.4–4.3) among the responders. For RBD-specific s-IgA, 57 of 466 vaccine recipients showed positive conversion (GMT 3.8 (95% CI 3.5–4.1) in responders). Only weak immunogenicity in peripheral blood was detected, which is consistent with previous observations in animals of a weaker immune response in the circulation than in the respiratory tract. The results of phase 3 trial confirmed the efficacy of a mucosal SARS-CoV-2 vaccine, regardless of age, prior vaccination, or the presence of underlying medical conditions [136]. The dNS1-RBD two-dose regimen exhibited an overall vaccine efficacy of 28.2% (95% CI 3.4–46.6) against confirmed symptomatic SARS-CoV-2 infection, regardless of prior immunization, when evaluated 15 days or more after vaccination (median follow-up: 161 days [IQR 111–189]). Short-term efficacy, specifically between 15 and 90 days, was found to be 32.6% (8.2–50.5). dNS1-RBD was the first intranasal vaccine against COVID-19, which obtained emergency use authorization in China in December 2022.

Delta NS technology was also used in the design of another vaccine, Corfluvec. Corfluvec contains two influenza virus-based vectors (H3N2 and H1N1pdm09) that carry a modified NS gene encoding the N protein of SARS-CoV-2. A phase 1,2 clinical trial assessing the safety and immunogenicity of this intranasal vaccine was carried out (NCT05696067) [137].

Thus, in clinical trial results, favorable safety and immunogenicity profiles of the vaccines listed above were reported. Nevertheless, further extensive large-scale research of their efficacy in human populations is clearly needed for combined vaccines’ implementation into the routine practice of influenza and COVID-19 medical management.

## 7. Conclusions and Future Perspectives

The co-circulation of COVID-19 and influenza highlights the potential for simultaneous outbreaks, particularly during peak respiratory illness seasons, which could significantly impact public health. This dynamic circulation raises concerns about the risk of infection with either virus. It poses a serious threat to global health and underscores the need for a contemporary infectious disease control, necessitating integrated diagnostic, preventive, and immunization strategies. Co-infection with influenza viruses and SARS-CoV-2 exacerbates respiratory disease, prolongs pneumonia, and increases mortality. Future studies should focus on further elucidating the mechanisms underlying the exacerbation of respiratory disease caused by co-infection, as well as the optimal strategies for co-administering influenza and COVID-19 vaccines.

Healthcare professionals should remain vigilant, monitoring both infections and promoting simultaneous or combination vaccination against COVID-19 and influenza. The main advantage of simultaneous vaccination is improved efficiency, allowing multiple vaccines to be administered at once. This approach reduces the number of visits, minimizing scheduling challenges and missed doses. In addition, it increases the vaccination rate and adherence to recommended vaccine schedules, contributing to higher community immunity and overall safety. Moreover, it can reduce healthcare costs by minimizing the resources required for multiple appointments, thereby improving the overall efficiency of vaccination programs. Importantly, when developing future vaccination strategies, global genomic surveillance as well as regulatory mechanisms should be harmonized to simplify the procedure for approving platforms and justifying the composition of seasonal dual-use vaccines.

In addition to influenza and SARS-CoV-2, other major respiratory pathogens, such as respiratory syncytial virus (RSV), should also remain the focus of the healthcare system, given the coincidence in the seasonality of their circulation. Taking this into account, triplet vaccine candidates’ development or combined administration strategies has become a significant trend. Recent clinical trials have confirmed the feasibility of triple vaccination against SARS-CoV-2, influenza and RSV. Particularly, Neutel et al. conducted a Phase 1/2 trial involving volunteers ≥65-year-olds (NCT05886777) to assess the safety, tolerability and immunogenicity of a combined vaccine for RSV and COVID-19 (RSVpreF + BNT162b2) when given alone or with a seasonal flu vaccine [138]. All vaccine groups studied were well tolerated, with noninferiority for all immunogenicity comparisons. In another recent Phase 3 trial (NCT05330975), authors studied a co-administration of RSV mRNA-1345 vaccine with SIIV4 vaccine, or RSV mRNA-1345 with SARS-CoV-2 mRNA-1273.214 vaccine among volunteers aged 50 or older. The co-administered vaccines revealed mostly non-inferior immune responses in comparison to individual vaccines and had admissible safety profiles [118]. Similarly, the U.S. Vaccine Adverse Event Reporting System (VAERS)-based analysis of influenza, COVID-19 and RSV vaccines co-administration in 2023–2024 supported an overall favorable co-vaccination safety profile in older adults, with most adverse events being non-serious and self-limiting [139].

Thus, co-vaccination against COVID-19, influenza, and RSV increases vaccination coverage, reduces the burden on the healthcare system, and increases convenience for the elderly. The CDC and other regulators consider it acceptable to administer all three vaccines in one visit, taking into account the patient’s individual risks and preferences.

To summarize, our review provides a comprehensive synthesis of current evidence spanning epidemiology, clinical outcomes, diagnostic methodologies, vaccination policies, and emerging vaccine technologies. We included in the complex analysis (i) the results of integrated genomic surveillance characterizing SARS-CoV-2 and influenza virus co-circulation and co-infection, (ii) highlighted the peculiarities of interferences viruses in experiments in vitro and in vivo. (iii) The focus of our review is experimental and clinical recent studies which have confirmed the effectivity and safety of simultaneous vaccination against SARS-CoV-2 and seasonal influenza. (iv) We paid special attention to the principal developments in different combined vaccine technologies against respiratory viruses, including influenza and SARS-CoV.

The COVID-19 pandemic was officially over in 2023, which inevitably made significant adjustments to epidemiological research and vaccine development. Particularly, the results of a significant part of the performed clinical trials have not been published, and we did not have the opportunity to summarize them. These points could be figured out as limitations of our review.

By consolidating global clinical data and national vaccination policies, we demonstrate that simultaneous vaccination constitutes a scientifically supported and operationally effective approach to enhance population-level protection, particularly among high-risk groups. Moreover, advances in combined and next-generation vaccine platforms highlight promising avenues toward simplified immunization schedules and improved pandemic preparedness. The growing body of clinical trials and real-world evidence indicates that co-administration of COVID-19 and influenza vaccines is safe, immunogenic, and operationally feasible, providing a practical strategy to enhance vaccine uptake. Importantly, this work underscores the need for coordinated surveillance systems, harmonized vaccination guidelines, and continued investment in translational vaccine research. Future studies should prioritize long-term real-world effectiveness, immunological durability, and optimization of simultaneous vaccination and combined vaccine formulations. Simultaneous and combined vaccination strategies should be evaluated across diverse populations and settings to inform public health policy and guide future pandemic preparedness. These efforts will be essential to strengthening public health resilience against the converging threats posed by seasonal influenza and emerging SARS-CoV-2 variants.

## Figures and Tables

**Table 1:** Animal studies of co-vaccination against SARS-CoV-2 and influenza virus.

Vaccine	Platform	Composition	Administration Route	Key Results	Ref.
PiCoVacc/Flu vaccine	Inactivated SARS-CoV-2 Split-virion influenza Vaccine	Inactivated SARS-CoV-2 virus Split-virion influenza virus	IP	Neutralizing antibodies Protection against SARS-CoV-2 and H1N1 infection	[53]
Quadruple microparticulate vaccine	Inactivated viruses encapsulated into PLGA polymer microparticles	Inactivated SARS-CoV-2 Delta and Omicron variants. Inactivated Influenza A H1N1 and H3N2 variants. AddaVax adjuvant	IN	Antigen-specific IgG (serum) and mucosal IgA (lung). Activation of cytotoxic (CD8+) and helper (CD4+) T-cells in lymph nodes and spleen.	[74]
Chimeric Influenza virus	Live attenuated or inactivated virus	Chimeric virus in live attenuated or inactivated form displaying influenza HA and SARS-CoV-2 RBD on its envelope	IN, IM	Neutralizing antibodies Protection from lethal challenge with both pathogens in mice.	[75]
Double-hit Flu-RBD vaccine	VLP Vaccine	SARS-CoV-2 RBD conjugated onto inactivated influenza A virus	IM	RBD-specific IgG2a and IgG1 Th1/Th2 balanced cellular immune response High protection efficacy against SARS-CoV-2 challenge in hamsters Strong neutralization activity against wild-type influenza A H1N1 inactivated virus in mice	[76]
Trivalent S/H1/N1 enveloped VLP	VLP vaccine	Full length SARS-CoV-2 S-protein, H1N1 hemagglutinin (H1) and neur aminidase (N1) co-incorporated into enveloped VLP SLA Archaeosome adjuvant	IM	Specific IgG to S, H1, N1, and cellular immune responses stimulation	[77]
Self-assembling SARS-CoV-2 RBD-trimer and Influenza H1N1 HA1-trimer	Recombinant protein vaccine	SARS-CoV-2-RBD-trimer and HA1-trimer Liposomal saponin-based MA103 adjuvant	IM	HAI for Influenza RBD-specific IgG Neutralizing antibody for SARS-CoV-2 Th1/Th2 balanced cellular immune response High protection efficacy against lethal SARS-CoV-2 and homogenous H1N1 influenza co-infection	[78]
Flu-COVID combo vaccine	Recombinant protein vaccine	Truncated H1 and H3 hemagglutinin (aa 1–528) and SARS-CoV-2 S protein (aa 1–1213) AddaVax adjuvant	IM	Neutralizing antibodies against both influenza and SARS-CoV-2 Specific IgGs against HA and S protein Protection from lethal challenge with both viruses in mice.	[79]
Flu-COVID pentavalent vaccine	Recombinant protein vaccine	SARS-CoV-2 RBD fused with the Fc fragment of the human IgG hemagglutinin surface antigens of the viruses A/H1N1-pdm09, A/H3H2, B/Yamagata, B/Victoria Betulin-based adjuvant	IM	RBD-specific and HA-specific IgG HAI for A/H1N1-pdm09, A/H3H2, B/Yamagata, B/Victoria viruses SARS-CoV-2 neutralization	[80]
AR-CoV/IAV	mRNA-LNP	LNP-encapsulated mRNA encoding HA from H1N1 and RBD from SARS-CoV-2 S protein	IM	Robust protective antibodies Antigen-specific cellular immune responses against SARS-CoV-2 and IAV Mice protection from coinfection with IAV and the SARS-CoV-2 Alpha and Delta variants	[81]
FLUCOV-10	mRNA-LNP	LNP-encapsulated mRNA encoding HA from H1N1 pdm09, H3N2, B/Victoria, B/Yamagata, H5N1, H7N9, S protein from four SARS-CoV-2 variants	IM	IgG antibodies, neutralizing antibodies, and antigen-specific cellular immune responses against all the vaccine-matched viruses of influenza and SARS-CoV-2 Complete protection in mouse models against both homologous and heterologous strains of influenza and SARS-CoV-2	[82]
QIV and BNT162b2	QIV: split-virion vaccine BNT162b2: mRNA-LNP	QIV: split-virion from 4 strains (A/H1N1, A/H3N2, B/Victoria, B/Yamagata) BNT162b2: LNP-encapsulated mRNA encoding SARS-CoV-2 S protein	IM	HAI and Binding Antibody Titers (HA, NA) Binding and Neutralizing Antibody Titers Complete protection in mouse models against lethal challenge with either virus	[83]
Influenza/COVID-19 Combination mRNA Vaccine	mRNA-LNP	LNP-encapsulated mRNA encoding: Influenza HA fusion proteins (dumbbell/trimer design with bacteriophage T4 foldon) from 4 strains (A/H1N1, A/H3N2, B/Victoria, B/Yamagata) SARS-CoV-2 RBD fusion protein (bivalent dumbbell).	IM	HAI antibodies titers against A/H1N1, A/H3N2, B/Victoria, B/Yamagata. - SARS-CoV-2-specific: Neutralizing antibody titers against XBB.1.5 variant.	[84]
AdC68-CoV/Flu	Vectored vaccine	Chimpanzee adenovirus 68 (AdC68) vector encoding SARS-CoV-2 RBD and H7N9 hemagglutinin	IM	Anti-H7 IgG Neutralizing antibody for SARS-CoV-2 Extensive RBD-specific T cell responses of splenocytes Protection against lethal SARS-CoV-2 challenge	[87]
AdC68-HATRBD	Vectored Vaccine	Chimpanzee adenovirus 68 (AdC68) vector encoding SARS-CoV-2 RBD Beta-Alpha chimeric dimer and Omicron-Delta chimeric dimer, numerous SARS-CoV-2 T cell epitopes, and full-length HA of H1N1 pdm09	IN	IgG, mucosal IgA, neutralizing antibodies, and memory T cells, protecting the mice from SARS-CoV-2 BA.5.2 and pandemic H1N1 infections.	[88]
Delta-19	Vectored Vaccine	Influenza Delta NS1 vector expressing key immunogenic proteins of SARS-CoV-2 and influenza viruses	IN	Neutralizing antibodies against SARS CoV-2 and Influenza viruses	[92]
FluVec-N	Vectored Vaccine	Influenza A/PR/8/34 (H1N1) vector carrying the SARS-CoV-2 N protein C terminal fragment (aa211–369) fused to the truncated NS1 gene	IN	Neutralizing antibody for SARS-CoV-2 N-Protein Specific antibodies in BAL Virus-specific effector and resident CD8+ lymphocytes in lungs Reduced weight loss and viral load in the lungs following infection with the SARS-CoV-2 beta variant.	[93]
dNS1-RBD Pneucolin	Vectored Vaccine	Live attenuated H1N1 pdm09 virus with NS1 gene replaced by SARS-CoV-2 RBD	IN	Local RBD-specific T cell response in the lung RBD-specific IgA and IgG response Attenuating pro-inflammatory cytokine levels post SARS-CoV-2 challenge, thereby reducing excess immune-induced tissue injury Cross-protection against H1N1 and H5N1 viruses	[94,95]
∆NA(RBD)-Flu	Vectored Vaccine	Influenza A/WSN/33 (H1N1) with H3 from A/Aichi/2/1968 and NA gene replaced by SARS-CoV-2 RBD	IN	Neutralizing antibodies against SARS-CoV-2 and Influenza virus	[96]
3×LAIV/CoV-2	Vectored Vaccine	Licensed trivalent LAIV with H1N1/H3N2 strains encoding conserved SARS-CoV-2 T-cell epitopes. B/Victoria strain is unmodified	IN	Serum antibodies specific to all three influenza strains Protection against challenge from either influenza strain and SARS-CoV-2 challenge. T-cell response to SARS-CoV-2 epitopes	[97]

Abbreviations: IM—intramuscular, IN—intranasal, IP—intraperitoneal, HA—hemagglutinin, HAI—hemagglutination inhibition, NA—neuraminidase, RBD—receptor-binding domain, Ig—Immunoglobulin, BAL—Bronchoalveolar lavage, LNP—Lipid Nanoparticles, PLGA—Co-polymer of lactic and glycolic acids.

**Table 2:** Parameters of clinical studies of influenza and COVID-19 vaccines co-administration.

Vaccine	Target	Manufacturer	Group of Participants (Age)	Type of Trial	Safety and Reactogenicity Profile	Immunogenicity	Ref.
NVX-CoV2373_ recombinant spike protein with matrix-M adjuvant	SARS-CoV-2	Novavax	Adult ≥ 18 (n = 15,187)	Clinical trial Phase 3	Safety–Local/Systemic Reaction–Adverse Events	HAI titers for Seasonal Influenza A and B strains Anti-SARS-CoV-2-spike IgG	[107]
Flucelvax Quadrivalent	Influenza	Seqirus					
Fluad							
COVID-19 mRNA booster vaccine	SARS-CoV-2	Pfizer-BioNTech	≥12 (n = 61,390)	Study on v-safe platform	Safety–Local/Systemic Reaction	N/A	[103]
SIV	Influenza	N/A					
COVID-19 mRNA booster vaccine	SARS-CoV-2	Moderna	≥12 (n = 30,633)				
SIV	Influenza	N/A					
mRNA-1273	SARS-CoV-2	Moderna	Adult ≥ 65 (n = 306)	Clinical trial Phase 2	Safety–Adverse Events	HAI titers for Seasonal Influenza A and B strains Anti-SARS-CoV-2-spike IgG	[104]
Fluzone QIV-HD	Influenza	Sanofi Pasteur					
BNT162b2 BA.4/5 bivalent_mRNA	SARS-CoV-2	Pfizer-BioNTech	Adult ≥ 18 (n = 3,442,996)	Retrospective comparative effectiveness study	Safety–Weighted hazard ratio	N/A	[105]
SIV	Influenza	N/A					
COVID-19 mRNA booster vaccine	SARS-CoV-2	N/A	Median: 48 years (n = 2449)	Vaccine Adverse Event Reporting System (VAERS)	Safety–Adverse Events	N/A	[106]
QIV	Influenza						
ChAdOx1	SARS-CoV-2	Pfizer-BioNTech	Adult ≥!8 years (n = 679)	Multicentre, randomized, controlled, phase 4 trial ConFluCov study	Safety–Local/Systemic Reaction–Adverse Events	HAI titers for Seasonal Influenza A and B strains Anti-SARS-CoV-2-spike IgG	[108]
BNT162b2		Pfizer-BioNTech					
Adjuvanted TIV (FluAd (MF59))	Influenza	Seqirus					
Flucelvax QIV		Seqirus					
Flublok Quadrivalent (QIVr)		Sanofi Pasteur					
BNT162b2mRNA	SARS-CoV-2	Pfizer-BioNTech	Adult (n = 1231)	Preference-based non-randomized controlled study	Safety	Anti-SARS-CoV-2-spike IgG	[112]
mRNA-1273	SARS-CoV-2	Moderna					
InfluvacTetra	Influenza	Abbott					
Omicron BA.4/BA.5–adapted bivalent_mRNA	SARS-CoV-2	Pfizer-BioNTech	Adult (n = 588)	Prospective cohort study	Safety–Local/Systemic Reaction–Adverse Events	Anti-SARS-CoV-2-spike IgG	[113]
Influvac Tetra	Influenza	Abbott					
BNT162b2_mRNA	SARS-CoV-2	Pfizer-BioNTech	Adult 18–65 (n = 1134)	Clinical trial Phase 3	Safety–Local/Systemic Reaction–Adverse Events	HAI for Seasonal Influenza A and B strains Anti-SARS-CoV-2-spike IgG	[114]
Afluria Quad	Influenza	Seqirus					
Inactivated SARS-CoV-2 vaccine (CoronaVac) + inactivated quadrivalent influenza vaccine (IIV4)	SARS-CoV-2 + Influenza	Sinovac Biotech	Adults 18–59 (n = 484)	Randomized, open-label, controlled clinical trial Phase 4	Safety–Local/Systemic Reaction–Adverse Events	Neutralizing antibodies against SARS-CoV-2	[115]
mRNA-1273	SARS-CoV-2	Moderna	Adult 18–75	Clinical trial Phase 1/2	Safety–Local/Systemic Reaction–Adverse Events	HAI for Seasonal Influenza A and B strains Titer of VAC62 Neutralizing Antibody for SARS-CoV-2	[116]
mRNA-1010	Influenza						
mRNA-1073	SARS-CoV-2/influenza						
Omicron XBB.1.5–containing COVID-19 mRNA booster vaccine (Spikevax)	SARS-CoV-2	Moderna	Adult (n = 56)	Open-label, randomized trial	Safety–Local/Systemic Reaction–Adverse Events	Neutralizing antibody, Anti-SARS-CoV-2-spike IgG	[117]
Afluria Quad	Influenza	Seqirus				HAI for Seasonal Influenza H1, H3, and B-Vic	
Afluria quadrivalent inactivated influenza vaccine + Moderna monovalent SARS-CoV-2 mRNA (XBB.1.5)	Influenza virus + SARS-CoV-2	Seqirus (Afluria) and Moderna	Adults ≥ 18 (n = 56)	Randomized controlled clinical trial	Safety–Local/Systemic Reaction–Adverse Events	HAI titers against seasonal influenza A(H1N1), A(H3N2), and B-Victoria strains; SARS-CoV-2–specific binding IgG and neutralizing antibody titers	[117]
mRNA-1345 (RSV) + SIIV4 or SARS-CoV-2 mRNA-1273.214 (bivalent)	Respiratory syncytial virus (RSV), Influenza A(H1N1), A(H3N2), B/Victoria, B/Yamagata, SARS-CoV-2 (ancestral D614G, Omicron BA.1)	Moderna	Adults ≥ 50 (n ≈ 3300)	Randomized, observer-blinded, placebo-controlled, Phase 3 clinical trial	Safety–Local/Systemic Reaction–Adverse Events	RSV-A neutralizing antibodies; HAI titers against influenza A(H1N1), A(H3N2), B/Victoria, and B/Yamagata strains; SARS-CoV-2 neutralizing antibodies against ancestral (D614G) and Omicron BA.1 variants	[118]
Quadrivalent inactivated influenza vaccines ± pneumococcal vaccine ± SARS-CoV-2 vaccine	Influenza virus + Streptococcus pneumoniae + SARS-CoV-2	N/A	Adults ≥ 60 (n = 105,527)	Population-wide retrospective cohort study	N/A	N/A	[119]
ARCT-2303 (self-amplifying mRNA COVID-19 vaccine) ± seasonal inactivated influenza vaccine	SARS-CoV-2 (Omicron XBB.1.5.6) + Influenza	Arcturus Therapeutics	Adults ≥ 18 (n = 1499)	Randomized, observer-blind, placebo-controlled, Phase 3 multicenter clinical trial	Safety–Local/Systemic Reaction–Adverse Events	Neutralizing antibodies against SARS-CoV-2 Omicron XBB.1.5.6; HAI titers against quadrivalent influenza vaccine strains	[120]
Ad26.COV2.S ± quadrivalent standard-dose (SD) or high-dose (HD) influenza vaccine	SARS-CoV-2 + Influenza	Janssen Vaccines and Prevention BV (Ad26.COV2.S); Seqirus (Afluria Quadrivalent, SD); Sanofi Pasteur (Fluzone High-Dose Quadrivalent, HD)	Adults ≥ 18 (n = 859)	Randomized, double-blind, Phase 3 non-inferiority clinical trial	Safety–Local/Systemic Reaction–Adverse Events	SARS-CoV-2 Spike-specific antibodies; HAI titres against influenza A(H1N1), A(H3N2), B/Victoria, and B/Yamagata strains	[121]
mRNA COVID-19 vaccine ± quadrivalent inactivated influenza vaccine (IIV4)	SARS-CoV-2 (D614G, BA.4/BA.5, XBB.1.5) + Influenza	N/A	Children and adults ≥ 5 (n = 335)	Randomized clinical trial	Safety	SARS-CoV-2 pseudovirus neutralizing antibody titers (D614G, BA.4/BA.5, XBB.1.5)	[122]

Abbreviations: HAI—hemagglutination inhibition, N/A—not available, SIV—seasonal influenza vaccine, TIV—trivalent influenza vaccine, QIV—quadrivalent influenza vaccine, QIV-HD—high-dose quadrivalent influenza vaccine, V-safe—vaccine safety. Note: in all cases, the vaccines were administered intramuscularly.

**Table 3:** Clinical trials of combined influenza and SARS-CoV-2 vaccines.

Vaccine	Manufacturer	Platform	Administration Route	Type of Trial	Ref.
Combined modified RNA COVID-19 and Influenza vaccine	Pfizer BioNTech	mRNA LNP	IM	Phase 3	[129]
Combined COVID-19 and Influenza mRNA 1073	Moderna	mRNA LNP	IM	Phase 1,2	[130]
Combined COVID-19 and Influenza mRNA 1083	Moderna	mRNA LNP	IM	Phase 1–3	[131,132]
mRNA Flu/COVID-19	GlaxoSmithKline	mRNA LNP	IM	Phase 1	[133]
qNIV/CoV2373	Novavax	Nanoparticle vaccine	IM	Phase 2	[134]
dNS1-RBD, Pneucolin	Beijing Wantai Biological Pharmacy Enterprise	Vector vaccine	IN	Phase 1–3	[135,136]
Corfluvec	Smorodintsev Research Institute of Influenza, Russia	Vector vaccine	IN	Phase 1,2	[137]

Abbreviations: LNP—lipid nanoparticles, IM—intramuscular, IN—intranasal.

## Data Availability

Data sharing is not applicable.

## Abbreviations

ACE2	Angiotensin-Converting Enzyme 2
AdC68	Chimpanzee adenovirus 68
BAL	Bronchoalveolar lavage
HA	Hemagglutinin
HAI	Hemagglutination Inhibition
IAV	Influenza A virus
Ig	Immunoglobulin
IM	Intramuscular
IN	Intranasal
IP	Intraperitoneal
IND	Investigational New Drug
LNP	Lipid Nanoparticle
PLGA	Co-polymer of lactic and glycolic acids
qIRV	Quadrivalent influenza modRNA vaccine
QIV	Quadrivalent Inactivated Influenza Vaccine
RBD	Receptor-Binding Domain
SARS-CoV-2	Severe Acute Respiratory Syndrome Coronavirus 2
SIV	Seasonal Influenza Vaccines
SIIV	Seasonal Inactivated Influenza Vaccine
TIV	Trivalent Inactivated Influenza vaccine
QIV-HD	High-dose quadrivalent influenza vaccine
V-safe	Vaccine safety
VAERS	Vaccine Adverse Event Reporting System
VLP	Virus-Like Particle
